# Effects of an Integrative Day Care Clinic Program with a Focus on Nature Therapy in a Hospital Park Setting on Quality of Life in Oncological Patients—A Non-Randomized Controlled Study

**DOI:** 10.3390/cancers15184595

**Published:** 2023-09-16

**Authors:** Lisa Kuballa, Christian S. Kessler, Farid I. Kandil, Christel von Scheidt, Meline Meinköhn, Barbara Koch, Manfred Wischnewsky, Andreas Michalsen, Michael Jeitler

**Affiliations:** 1 Institute of Social Medicine, Epidemiology and Health Economics, Charité—Universitätsmedizin Berlin, Corporate Member of Freie Universität Berlin and Humboldt-Universität zu Berlin, 10117 Berlin, Germany; christian.kessler@charite.de (C.S.K.); farid-ihab.kandil@charite.de (F.I.K.); meline.meinkoehn@charite.de (M.M.); andreas.michalsen@charite.de (A.M.); michael.jeitler@charite.de (M.J.); 2 Department of Internal Medicine and Nature-Based Therapies, Immanuel Hospital Berlin, 14109 Berlin, Germany; christel.vonscheidt@immanuelalbertinen.de (C.v.S.); barbara.koch@immanuelalbertinen.de (B.K.); 3 Department of Mathematics and Computer Science, University Bremen, 28359 Bremen, Germany; wischnewsky@escience.uni-bremen.de

**Keywords:** cancer, day care, integrative medicine, lifestyle, MICOM, Mind–body Medicine, mindfulness, meditation, nature, oncology

## Abstract

**Simple Summary:**

Cancer often causes long-term physical and psychological impairments. Lifestyle modification and nature-based interventions (NBIs) can have a positive impact on patients’ quality of life (QOL). However, there is sparse scientific research on the effects of natural environments in the treatment of cancer patients. Therefore, we integrated intensified nature experiences into the scientifically substantiated therapy approach of an oncology day care clinic program. This study aimed to compare the effects of a nature-based oncology day care clinic intervention to conventional oncology day care clinic intervention. A positive impact of the day care clinic program on QOL, fatigue, and psychological parameters in cancer patients was shown. In addition, NBIs seem to have more pronounced effects, which need to be further proven.

**Abstract:**

Cancer often causes long-term physical and psychological impairments. Lifestyle modification and nature-based interventions (NBIs) can have a positive impact on patients’ quality of life (QOL). This participants-blinded, non-randomized controlled study assessed parameters at weeks 0, 12, and 24, including, as a primary endpoint, QOL in cancer patients on the Functional Assessment of Cancer Therapy—General (FACT-G) at week 12. QOL in breast cancer patients, fatigue, well-being, stress, anxiety/depression, socio-psychological well-being, benefits of nature interaction, insomnia, self-efficacy, mindfulness, and self-compassion were assessed as secondary endpoints. N = 107 cancer patients (96.3% women; 52.5 ± 9.3 years, 80.4% breast cancer) were assigned to either a 12-week nature-based (NDC; n = 56) or conventional (DC; n = 51) oncology day care clinic program, whereby the assignment group was not known to the participants. There was no significant group difference for the primary endpoint. At week 24, QOL, fatigue, mindfulness and self-compassion scores were significantly higher, and at weeks 12 and 24, the insomnia score was significantly lower in NDC compared to DC. In conclusion, this study indicates positive and clinically relevant effects of the program on QOL, fatigue, and psychological parameters. NBIs seem to have a more pronounced effect.

## 1. Introduction

Cancer is one of the most common causes of morbidity and mortality in industrialized societies [1]. In addition to disease- and treatment-related symptoms, cancer patients often suffer from psychological and physical complaints, even after completion of primary therapy. Fatigue, depression, and anxiety, as well as disturbed sleep, may lead to impaired quality of life and mood [2]. 

Complementary and integrative medicine (CIM) practices are widely used by cancer patients, mainly to reduce treatment-related side effects, e.g., to relieve pain and to strengthen their immune system [3,4,5]. In view of rising survival rates [6], the need for treatment options that improve disease-related symptoms such as pain or fatigue is growing and gaining importance in oncology. Therefore, “integrative” oncology, which combines “conventional” cancer therapy with evidence-based CIM methods, is an emerging field [7,8]. 

Mind–body medicine (MBM) is an important method within CIM, where “practices […] focus on the interactions between the brain, mind, body, and behavior” [9]. In a broader sense, MBM also comprises health-related lifestyle issues to improve patients’ self-care and self-efficacy [9]. 

MBM practices have been studied in cancer patients in randomized, controlled clinical trials, which have showed positive effects on quality of life, pain intensity and pain coping, fatigue, and psychological well-being, including depression and anxiety [10,11].

It should be noted that the above studies evaluated unimodal interventions in clinical trials and usually recruited highly selected populations. We combined these MBM methods in an oncology day care clinic program and made it available for a general population of cancer patients. Cohort studies conducted at our department [12] and at the Department of Integrative Medicine, Evangelische Kliniken Essen-Mitte [13,14,15] have already evaluated this treatment approach. Improvements in quality of life, fatigue, and psychological parameters, especially anxiety and depression were shown [12,13,14,15]. Several compulsory health insurance funds cover the costs of this integrative oncology day care clinic program in Germany.

Furthermore, in recent years increasing amounts of evidence have been found for the health benefits of nature experiences and nature-based interventions (NBIs); among other things, positive effects on general well-being, autonomic nervous system functions, blood pressure, endocrine activity, and immune activity have been demonstrated [16,17,18]. Early findings from an intervention study on psychotherapy for depressive patients indicate that conducting therapy in nature (a forest environment) is more effective than conducting it in hospital [19]. However, there is sparse scientific research on the effects of natural environments in the treatment of cancer patients. 

Therefore, we integrated intensified nature experiences into the scientifically substantiated therapy approach of an MICOM (Mind–body Medicine in Integrative and Complementary Medicine) based oncology day care clinic program. This study aimed to assess the effects of a nature-based oncology day care clinic intervention for cancer patients on patient-reported outcomes (PROs), compared to a conventional oncology day care clinic intervention. 

## 2. Materials and Methods

### 2.1. Design

This participants-blinded, non-randomized controlled study was approved by the ethics committees of the Charité—Universitätsmedizin Berlin (EA4/026/20). After registration at ClinicalTrials.gov (NCT04411251), reporting was performed according to the CONSORT (Consolidated Standards of Reporting Trials) extension for pilot and feasibility trials [20]. 

### 2.2. Participants

Patients were recruited in the study department, checked for eligibility and assessed by a study physician. If all inclusion criteria and no exclusion criteria were met and informed consent was signed, patients were enrolled in the study. The participants were assigned to the group with the next possible start date of the day care clinic program, as there is usually a waiting time of several weeks for these clinics, due to high demand. The group they were assigned to was not known to the participants.

Adult patients between the ages of 18 and 85 years, diagnosed with any type of cancer were included. Exclusion criteria included (1) cognitive impairment that would interfere with answering questionnaires or the intervention, (2) pre-existing severe mental disorders, (3) pregnancy or breastfeeding, or (4) participation in another study.

### 2.3. Nature-Based Oncology Day Care Clinic Program (NDC)

Participants took part in a 12-week nature-based day care clinic program with weekly 6-h sessions, of which 1.5 h were in nature. A semi-residential format was chosen to improve group cohesion and the patient–therapist relationship. The day care clinic program was based on the mindfulness-based stress reduction (MBSR) program and the Mindbody medicine cancer program of the Benson–Henry Institute for Mind–Body Medicine, which was further developed according to the MICOM model [9]. The focus was on relaxation techniques, rooted in psychoneuroendocrinology, and used meditation, gentle yoga, breathing exercises, muscle relaxation, physical exercise, cognitive restructuring, and social support. In addition, CIM methods of self-care were incorporated, such as nutrition, self-massage, and hydrotherapy. Patients were strongly encouraged to use self-help techniques to enhance self-empowerment. The aim of the program was to reduce disease and treatment induced stress, to improve health-related lifestyles, coping skills, and acceptance of illness. MSc- or Ph.D.-level health professionals specially trained in MBM and psychosocial counseling held the day care clinic program. Moreover, weekly rounds were made by physicians, in which patients received individual recommendations for support. Therapists were trained to instruct with a mindful attitude during the entire program. 

NBI training took place in an area close to nature, especially in the hospital park of the Immanuel Hospital Berlin, Berlin, Germany. These included relaxation techniques guided directly in nature, gentle movement exercises such as yoga and Qigong in nature as well as nature awareness and nature meditation exercises. Furthermore, participants were asked to spend at least 60 min a day in nature and to practice the exercises taught in the day care clinic close to nature.

### 2.4. Conventional Oncology Day Care Clinic Program (DC)

This intervention was the same as that of the NDC group, but took place mainly in the hospital building. Participants were asked to practice the above-mentioned exercises, taught in the day clinic, at home for at least 60 min a day.

### 2.5. Outcome Measures

Assessment of the following PROs took place at baseline and at 12 and 24 weeks after enrollment.

- The primary endpoint was the difference between changes from baseline to week 12 in the quality of life in cancer patients on the Functional Assessment of Cancer Therapy—General (FACT-G) scale, including 4 domains of health-related quality of life: physical (7 items), social (7 items), emotional (6 items), and functional (7 items) well-being [21].
- Quality of life assessment in breast cancer patients on the Functional Assessment of Cancer Therapy—Breast Cancer (FACT-B) consisted of the 27-item FACT-G and the 10-item breast cancer subscale (BCS) that addressed additional concerns associated with breast cancer and its treatment [22].
- Fatigue assessment on the Functional Assessment of Chronic Illness Therapy—Fatigue (FACIT-F) consisted of the 27-item FACT-G and the 13-item fatigue subscale, (FS) [23], measuring self-reported fatigue and its impact upon daily activities and function is.
- Well-being on the 5-item WHO-Five Well-Being Index (WHO-5) [24].
- Stress on the 10-item Perceived Stress Scale (PSS-10) for measuring perceived stress within the last month [25].
- Anxiety and depression on the 14-item Hospital Anxiety and Depression Scale (HADS-D) for assessing anxiety and depression symptoms [26].
- Socio-psychological well-being on the 8-item Flourishing Scale (FS-D) [27]. Flourishing of the personality refers to the self-assessment of one’s own perspective,, potential and resources [27].
- Perceived benefits of interacting with nature on the 11-item Perceived Benefits of Nature Questionnaire (PBNQ) [28].
- Insomnia on the 7-item Insomnia Severity Index (ISI) for assessing the nature, severity, and impact of insomnia [29].
- Self-efficacy on the 3-item Self-Efficacy Scale—Short Form (ASKU) [30]. Self-efficacy reflects “individual competence expectations to be able to deal with difficulties and obstacles in daily life“ [30].
- Mindfulness on the 14-item Freiburg Mindfulness Inventory (FMI) [31].
- Self-compassion on the 26-item Self-Compassion Scale (SCS-D) [32].

Evaluation, adherence to relaxation exercises and lifestyle (diet, Kneipp hydrotherapy, phytotherapy, relaxation exercises, cigarette/alcohol consumption, and sick leave) were assessed by questionnaires designed by the authors. A 5-point Likert scale was used to evaluate the day care clinic program and nominal/ordinal scales and duration in minutes were allocated as free text for the assessment of lifestyle-related issues. Participants completed a weekly review during the day care clinic program, in which they reflected on their exercise practice at home. Unfortunately, there were too many pieces of missing data, making analysis of this component impossible. Adverse effects were recorded using standardized forms.

### 2.6. Sample Size Calculation and Statistical Analysis

Based on a pre-study, it was determined that a total of 30 patients per group were required to detect a difference between groups at alpha = 0.05 and a statistical power of 80% (beta = 0.20) [12]. Accounting for a potential dropout rate of 15%, at least 72 participants needed to be included. However, due to good recruitment despite the Covid-19 pandemic, and since the program was part of the hospital’s regular care, we decided to enroll more patients. All analyses were conducted according to the intention-to-treat method, i.e., all participants were analyzed in the group to which they were originally assigned. Missing data were multiply imputed by Markov Chain Monte Carlo (MICE) methods [33,34], resulting in a total of 101 complete data sets.

For data analysis, differences between test results for V1 and V0, and for V2 and V0, were calculated. Test statistics are given as means ± standard deviations (M ± SD). Within-group differences were analyzed by paired *t*-tests and group differences by independent *t*-tests. Only the between-group test for the primary endpoint was tested in a confirmatory way, against an alpha = 0.05. All other comparisons within and between groups were conducted on an exploratory level only. Thus, no alpha adjustment (Bonferroni correction) was applied. ANCOVAs were used to test whether sociodemographic parameters such as sex, age, marital status, highest level of education, employment status, and self-reported monthly income, as well as the self-reported stress level (PSS at visit V0), had an impact on the primary endpoint. All analyses were performed using Statistical Package for Social Sciences software (IBM SPSS Statistics for Windows, Version 28.0. IBM Corp, Armonk, NY, USA). 

## 3. Results

### 3.1. Participants

**Figure 1:**
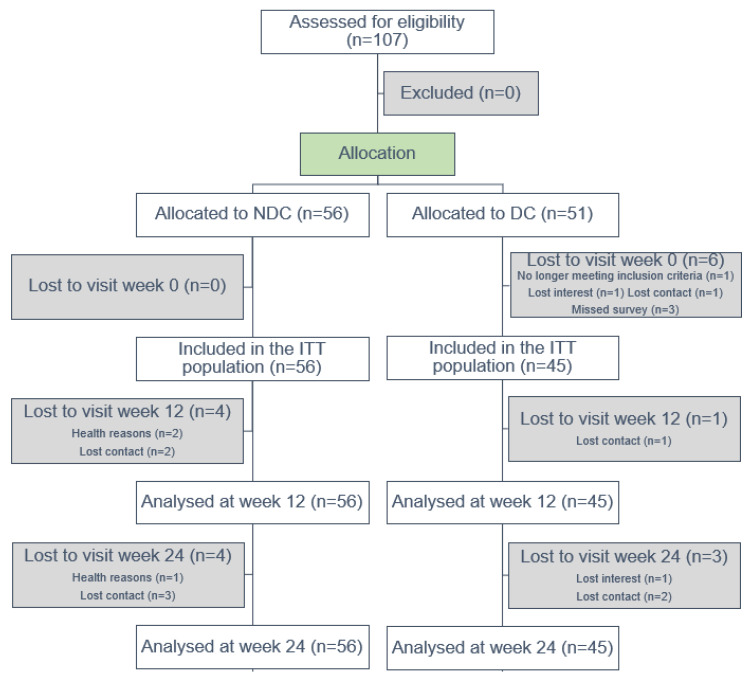
Study flow chart. Abbreviations: NDC, nature-based oncology day care clinic program; DC, conventional oncology day care clinic program; ITT, intention-to-treat.

A total of 107 patients were screened, enrolled after giving informed consent, and allocated to either the NDC (n = 56) or DC group (n = 51). Eleven participants (four in the NDC group and seven in the DC group) dropped out of the study before the primary endpoint in week 12. Another seven (four in the NDC group and three in the DC group) were missing at the follow-up in week 24 (Figure 1). Patients were recruited between July 2020 (first patient in) and October 2021. The last follow-up assessment was completed in May 2022 (last patient out). Participants’ characteristics are shown in Table 1. Of the total of 107 participants (mean age 52.5 ± 9.3 years), almost all (96.3%) were female, while about two-thirds had a university degree. Participants had a BMI of 23.9 ± 4.1 kg/m^2^. Eighty percent were diagnosed with breast cancer. More than half reported sleep disturbances and fatigue, and polyneuropathy was mentioned by 29% of the participants. Co-morbidities are listed in Table S1 in the Supplementary Material. 

### 3.2. Primary Endpoint

**Figure 2:**
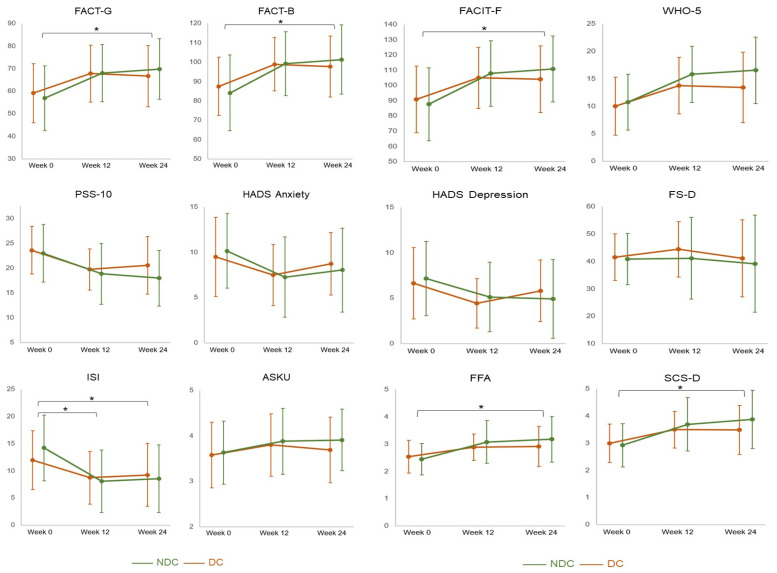
Multiplot on patient-reported outcomes. Legend: ASKU, Self-Efficacy Scale—Short Form; FACIT-F, Functional Assessment of Chronic Illness Therapy—Fatigue; FACT-B, Functional Assessment of Cancer Therapy—Breast Cancer; FACT-G, Functional Assessment of Cancer Therapy—General; FMI, Freiburg Mindfulness Inventory; FS-D, Flourishing Scale; HADS-D, Hospital Anxiety and Depression Scale; ISI, Insomnia Severity Index; NDC, nature-based oncology day care clinic program; DC, conventional oncology day care clinic program; PSS-10, Perceived Stress Scale; SCS-D, Self-Compassion Scale; WHO-5, WHO-Five Well-Being Index; * = *p* < 0.05. The error bars indicate standard deviations.

In respect of the primary endpoint, the FACT-G score, patients in both groups improved between the baseline measure and their visit at week 12 (NDC Δ = 11.0 ± 9.5, *p* < 0.0001; DC Δ = 8.7 ± 10.6, *p* < 0.0001). However, the hypothesized difference between groups did not become significant (Δ = 2.3 ± 2.0, *p* = 0.2573), and only the follow-up testing in week 24 yielded an (exploratively) significant group difference (Δ = 5.3 ± 2.2; *p* = 0.0161) (Table 2 and Figure 2).

### 3.3. Secondary Endpoints

From week 0 to week 12, within-group improvements in the range of moderate to large effects were obtained for most self-reported outcomes, i.e., quality of life (FACT-G and FACT-B), fatigue (FACIT-F), well-being (WHO-5), perceived stress (PSS-10), anxiety and depression (HADS), insomnia (ISI), self-efficacy (ASKU), mindfulness (FMI), and self-compassion (SCS-D). However, regarding between-group comparisons after 12 weeks, only the ISI score was significantly lower in NDC than in DC (Δ = 2.9 ± 1.1; *p* = 0.0116).

Positive within-group effects found after 12 weeks also persisted to week 24. In contrast to the comparisons for week 12, at week 24, more between-group comparisons became significant, with results indicating a better quality of life (FACT-G: Δ = 5.3 ± 2.2; *p* = 0.161 and FACT-B: Δ = 7.0 ± 2.8; *p* = 0.0129), less fatigue (FACT-F: Δ = 10.0 ± 4.1; *p* = 0.0162), higher mindfulness (FMI: Δ = 0.4 ± 0.2; *p* = 0.0298) and self-compassion (SCS-D: Δ = 0.5 ± 0.2; *p* = 0.0223), and less insomnia (Δ = 2.9 ± 1.1; *p* = 0.0108) in the NDC than in the DC group at the follow-up (Table 2 and Figure 2).

### 3.4. Covariates

The initial self-reported stress level (PSS at V0) was revealed to be a significant covariate for the primary endpoint. Higher initial stress levels (as indicated by higher values in the PSS) were significantly positively correlated with higher gains (differences) in the FACT-G for both comparisons, V1 vs. V0 (*p* < 0.001) and V2 vs. V0 (*p* < 0.001), indicating that patients with a higher initial stress level gained more from the intervention.

Apart from that, none of the other tested parameters (sex, age, marital status, highest level of education, employment status, and self-reported monthly income, as well as the self-reported stress level) had a significant impact on the FACT-G (primary endpoint).

### 3.5. Lifestyle

Results for lifestyle measures are listed in Table S2 (Supplementary Material). Patients’ diets changed slightly toward a plant-based diet during the study period. At baseline, participants used Kneipp hydrotherapy on average on one day, compared to three days at the follow-ups. The percentage of participants using phytotherapy increased from about 80% at baseline to nearly 100% post-intervention. Relaxation exercises were already practiced by about half of the participants at the beginning of the study, by almost all after 12 weeks and by about three quarters after 24 weeks. At baseline, participants in the NDC group exercised on average for 13.3 ± 34.5 and in the DC group for 7.6 ± 11.2 min per day, compared to 22.1 ± 20.9 and 20.2 ± 17.8 min per day after the intervention. Cigarette and alcohol consumption decreased overall after the intervention. The same applies to the number of sick days.

### 3.6. Evaluation

In all assessments, participants rated the effectiveness of the day care clinic program as strong. Most of them reported concerns about the implementation of the program before the start, but these negative expectations were not confirmed in the follow-ups (Table S3 in the Supplementary Material).

### 3.7. Adverse Events

No serious adverse events occurred that could be associated with the intervention. In the DC group, two patients suffered from a form of breast cancer recurrence during the course of the study and had to undergo surgery. Single participants reported restlessness and tension during certain exercises. Overall, 29 minor adverse events occurred in 19 patients in the DC group, and 18 in 13 patients in the NDC group (Table S4 in the Supplementary Material).

## 4. Discussion

This study aimed to assess the effects of a nature-based day care clinic intervention, compared to a conventional day care clinic intervention, on self-reported outcomes in cancer patients. Although the primary endpoint did not reach statistical difference between the groups, after the day care clinic intervention, we found (exploratively significant) improvements in both groups for almost all outcome measures, namely quality of life, fatigue, well-being, stress, anxiety, depression, insomnia, self-efficacy, mindfulness, and self-compassion. At week 12, the insomnia score (ISI) was significantly lower, and at week 24, quality of life (FACT-G, FACT-B), fatigue (FACIT-F), mindfulness (FMI) and self-compassion (SCS-D) were significantly higher and insomnia (ISI) significantly lower in the nature-based group.

With more than 90% women and about 80% breast cancer patients, the groups were similar regarding gender and cancer types. In total, a larger proportion had a higher level of education. At the start of the study, the entire population had lower levels of quality of life and increased stress, anxiety and insomnia levels.

The day care clinic intervention reduced stress and anxiety in both groups. Furthermore, participants had a higher level of quality of life at the end of intervention as well as at the 24-week follow-up. This is in line with the pre-study [12] and other findings that showed improvements in stress, anxiety, and depression through MBSR intervention [35,36,37]. Also, yoga has been shown in meta-analyses to improve quality of life and reduce anxiety, depression, stress, and fatigue, especially in patients with breast cancer [38,39]. In addition meta-analyses on psychosocial interventions found positive effects on health-related quality of life, mental health, and possibly survival in patients with prostate cancer [40] or breast cancer [41,42], but not in patients with head and neck cancer [43]. Psychosocial factors also have a major impact on the quality of life of patients with ovarian cancer [44]. Furthermore, for cancer patients of reproductive age, fertility preservation and family planning are important issues that affect quality of life [45,46,47].

The 24-week follow-up indicated that NDC significantly improved quality of life and fatigue in the between-group comparison. Recent studies have shown that NBIs can enhance health-related quality of life and mental health, particularly depressed mood, anxiety, and affect [48,49,50]. A natural environment is also associated with lower levels of stress hormones and higher levels of serotonin and endogenous opioids, which also contribute to better vitality and mood [51].

For several parameters, cut off values for clinically relevant effects have previously been reported. The minimum clinically important difference (MCID) for the FACT-G is defined as a value of 5, for the FACT-B as a value of 7, and for the FS as a value of 3. In our study the increases were greater than 7 points, 10 points, and 5 points, respectively [52,53]. With more than 6 points’ difference, the NDC group achieved the MCID for ISI at the 12-week visit [54]. Our study population experienced improvements in self-efficacy, mindfulness, and self-compassion through the MBM intervention. These parameters could interact with each other. In addition, a meta-analysis showed overall positive effects of mindfulness in natural environments, suggesting the important role of setting in the benefits of mindfulness-based interventions [55].

### Study Limitations

Our study has several limitations. First, we could not randomize the participants, because the study was conducted in the framework of a regular care setting at the day care clinic. Therefore, the clinic personnel were not blinded either. The participants were assigned to the group with the next possible start date of the day care clinic program, as these usually have a waiting time of several weeks, due to high demand. Because of the non-randomized design, group comparisons are limited. However, participants did not know whether they were assigned to the NDC or the DC group, so that non-specific effects, e.g., due to disappointment effects, were minimized.

Second, the DC group was smaller and the clinical characteristics of the groups were not completely balanced. Another limiting fact is that our study population consisted of predominantly female participants with a higher level of education. We aimed to include more men and patients with other types of cancer, but only a limited number of these patients were referred during the recruitment phase.

The fact that the majority of participants were diagnosed with breast cancer also limits the generalizability of the study results. Future studies in the field should focus on single cancer types to capture the impact of the interventions more accurately.

Another limitation is the mix of patients undergoing chemotherapy, radiotherapy, hormone therapy and surgery. Patients’ concerns may vary significantly in these different oncology settings. However, the physicians gave the patients individual recommendations to support them.

Another limiting factor is that the interventions were a mixture of different techniques that could not all be applied to participants in the same way and were not used in the same way by the participants. The sample size should have been larger than calculated, to account for variability in interventions, cancer types, and cancer treatments.

Also, adherence to each intervention component (e.g., mindfulness, nature interventions, diet, hydrotherapy, phytotherapy) could be assessed in more detail.

## 5. Conclusions

In summary, the results of this study indicate positive and clinically relevant effects of the day care clinic program on quality of life, fatigue, and psychological parameters. In addition, nature-based interventions seem to have more pronounced effects, which need to be further proven in randomized trials.

## Figures and Tables

**Table 1:** Sociodemographic and clinical characteristics at baseline.

	Total (n = 107)	NDC (n = 56)	DC (n = 51)
**Sociodemographic characteristics**			
Gender female n (%)	103 (96.3)	55 (98.2)	48 (94.1)
Age years (mean ± SD)	52.5 ± 9.3	53.2 ± 8.8	51.7 ± 9.9
**Marital status** n (%)			
Single	26 (25.7)	12 (21.4)	14 (31.1)
In a relationship	15 (14.9)	9 (16.1)	6 (13.3)
Married	46 (45.5)	25 (44.6)	21 (46.7)
Divorced or Separated	13 (12.9)	9 (16.1)	4 (8.9)
Widowed	1 (1.0)	1 (1.8)	0 (0.0)
**Highest level of education** n (%)			
University	66 (65.3)	35 (62.5)	31 (68.9)
Apprenticeship	19 (18.8)	16 (28.6)	3 (6.7)
A level	8 (7.9)	1 (1.8)	7 (15.6)
High school	8 (7.9)	4 (7.1)	4 (8.9)
**Employment status n** (%)			
Full-time	29 (28.7)	15 (26.8)	14 (31.1)
Part-time	20 (19.8)	10 (17.9)	10 (22.2)
Occasional	9 (8.9)	6 (10.7)	3 (6.7)
On sick leave	27 (26.7)	17 (30.4)	10 (22.2)
Retired	13 (12.9)	8 (14.3)	5 (11.1)
Unemployed	3 (3.0)	0 (0.0)	3 (6.7)
**Self-reported monthly income** n (%)			
<1000 €	13 (14.6)	6 (12.5)	7 (17.1)
1001−1500 €	16 (18.0)	8 (16.7)	8 (19.5)
1501−2000 €	14 (15.7)	9 (18.8)	5 (12.2)
2001−3000 €	32 (36.0)	15 (31.3)	17 (41.5)
3001−4000 €	10 (11.2)	9 (18.8)	1 (2.4)
>4000 €	4 (4.5)	1 (2.1)	3 (7.3)
**Clinical characteristics** (mean ± SD)			
Body mass index (kg/m^2^)	23.9 ± 4.1	23.8 ± 4.2	24.0 ± 3.9
Clinical systolic BP (mmHg)	121.1 ± 15.8	119.2 ± 16.8	123.0 ± 14.7
Clinical diastolic BP (mmHg)	78.7 ± 10.3	77.1 ± 10.3	80.2 ± 10.2
**Cancer type** n (%)			
Breast cancer	86 (80.4)	45 (80.4)	41 (80.4)
Stage of breast cancer			
Stage 0	8 (10.4)	4 (9.8)	4 (11.1)
Stage I	22 (28.6)	13 (31.7)	9 (25.0)
Stage II	36 (46.8)	18 (43.9)	18 (50.0)
Stage III	8 (10.4)	5 (12.2)	3 (8.3)
Stage IV	3 (3.9)	1 (2.4)	2 (5.6)
Unknown	9 (11.7)	4 (9.8)	5 (13.9)
Endometrial cancer	3 (2.8)	0 (0.0)	3 (5.9)
Ovarian cancer	3 (2.8)	2 (3.6)	1 (2.0)
Other cancer	15 (14.0)	9 (16.1)	6 (11.8)
**Treatment history** n (%)			
Surgery	97 (90.7)	51 (91.1)	46 (90.2)
Chemotherapy	65 (60.7)	35 (62.5)	30 (58.8)
Radiotherapy	67 (62.6)	33 (58.9)	34 (66.7)
Hormonal therapy	64 (59.8)	36 (64.3)	28 (54.9)
Continuing hormonal therapy	61 (57.0)	35 (62.5)	26 (51.0)
Targeted therapy	22 (20.6)	10 (17.9)	12 (23.5)
Continuing targeted therapy	12 (11.2)	5 (8.9)	7 (13.7)

Abbreviations: NDC, nature-based oncology day care clinic program; DC, conventional oncology day care clinic program; SD, standard deviation.

**Table 2:** Outcome measures (mean ± standard deviation). Bold *p*-values indicate significant group differences (*p* < 0.05).

		Week 0	Week 12							Week 24						
Outcome	Group	Mean ± SD	Mean±SD	Δ Mean ± SD_within_	P_within_	Cohen’s d_within_	Δ Mean ± SD_between_	P_between_	Cohen’s d_between_	Mean ± SD	Δ Mean ± SD_within_	P_within_	Cohen’s d_within_	Δ Mean ± SD_between_	P_between_	Cohen’s d_between_
FACT-G	NDC	56.9 ±14.3	68.0 ± 12.6	11.0 ± 9.5	<0.01	1.15	2.3 ± 2.0	0.26	0.23	69.8 ± 13.5	12.9 ± 11.0	<0.01	1.17	5.3 ± 2.2	**0.02**	0.49
	DC	59.1 ± 13.1	67.8 ± 12.6	8.7 ± 10.6	<0.01	0.82				66.7 ± 13.5	7.5 ± 10.7	<0.01	0.70			
FACT-G Physical well-being	NDC	18.1 ± 5.6	22.8 ± 4.8	4.7 ± 4.0	<0.01	1.16	1.0 ± 0.8	0.22	0.25	23.5 ± 5.0	5.4 ± 5.0	<0.01	1.07	1.6 ± 1.0	0.11	0.32
	DC	19.0 ± 5.0	22.6 ± 4.1	3.6 ± 4.3	<0.01	0.85				22.7 ± 4.2	3.8 ± 5.0	<0.01	0.75			
FACT-G Social well-being	NDC	13.0 ± 5.0	13.7 ± 5.2	0.7 ± 3.9	0.16	0.19	0.2 ± 0.7	0.74	0.06	14.3 ± 5.1	1.3 ± 4.3	0.02	0.31	1.6 ± 0.8	0.05	0.39
	DC	13.7 ± 4.7	14.2 ± 4.9	0.5 ± 3.0	0.25	0.17				13.4 ± 5.1	−0,2 ± 3.8	0.67	0.06			
FACT-G Emotional well-being	NDC	17.4 ± 4.0	20.7 ± 2.8	3.2 ± 3.3	<0.01	0.99	1.1 ± 0.6	0.07	0.35	20.6 ± 3.2	3.1 ± 4.3	<0.01	0.73	1.2 ± 0.7	0.11	0.31
	DC	17.8 ± 3.5	19.9 ± 2.8	2.1 ± 2.8	<0.01	0.75				19.7 ± 3.2	2.0 ± 3.0	<0.01	0.66			
FACT-G Functional well-being	NDC	8.4 ± 4.4	11.2 ± 4.2	2.8 ± 3.6	<0.01	0.79	0.3 ± 0.8	0.73	0.07	12.1 ± 4.4	3.6 ± 4.0	<0.01	0.91	1.4 ± 0.8	0.08	0.36
	DC	8.7 ± 4.6	11.2 ± 4.5	2.5 ± 4.0	<0.01	0.64				11.0 ± 4.5	2.2 ± 3.7	<0.01	0.60			
FACT-B	NDC	84.1 ± 19.7	99.216.5	15.1 ± 12.9	<0.01	1.17	3.7 ± 2.6	0.16	0.28	101.4 ± 17.9	17.2 ± 14.0	<0.01	1.23	7.0 ± 2.8	**0.01**	0.51
	DC	87.5 ± 15.2	98.9 ± 13.8	11.4 ± 13.5	<0.01	0.84				97.7 ± 15.7	10.2 ± 13.6	<0.01	0.75			
BCS	NDC	27.2 ± 7.7	31.3 ± 7.6	4.1 ± 6.9	<0.01	0.60	1.4 ± 1.4	0.31	0.20	31.5 ± 7.3	4.4 ± 6.2	<0.01	0.71	1.7 ± 1.3	0.22	0.21
	DC	28.4 ± 7.1	31.1 ± 6.4	2.7 ± 7.0	0.01	0.38				31.1 ± 6.6	2.7 ± 7.1	0.01	0.38			
FACIT-F	NDC	87.6 ± 23.9	107.9 ± 21.5	20.3 ± 18.1	<0.01	1.12	6.2 ± 3.7	0.10	0.33	110.9 ± 21.7	23.2 ± 21.4	<0.01	1.09	10.0 ± 4.1	**0.02**	0.49
	DC	90.8 ± 21.9	105.0 ± 20.2	14.1 ± 18.9	<0.01	0.75				104.1 ± 21.9	13.3 ± 19.5	<0.01	0.68			
FS	NDC	30.7 ± 11.3	40.0 ± 10.4	9.3 ± 10.4	<0.01	0.90	3.9 ± 2.0	0.05	0.39	41.0 ± 10.0	10.4 ± 12.0	<0.01	0.86	4.6 ± 2.2	**0.04**	0.41
	DC	31.7 ± 11.2	37.2 ± 9.4	5,4 ± 9,4	<0.01	0.58				37.4 ± 10.0	5.7 ± 10.4	<0.01	0.55			
WHO-5	NDC	10.7 ± 5.1	15.8 ± 5.1	5.1 ± 5.3	<0.01	0.96	1.3 ± 1.0	0.21	0.25	16.5 ± 6.1	5.8 ± 6.7	<0.01	0.86	2.4 ± 1.3	0.08	0.36
	DC	10.0 ± 5.3	13.8 ± 5.1	3.8 ± 5.2	<0.01	0.73				13.4 ± 6.4	3.4 ± 6.4	<0.01	0.54			
PSS	NDC	23.0 ± 5.8	18.9 ± 6.1	4.1 ± 6.2	<0.01	0.67	0.2 ± 1.1	0.82	0.04	18.0 ± 5.6	5.0 ± 6.6	<0.01	0.76	2.0 ± 1.3	0.12	0.31
	DC	23.6 ± 4.8	19.7 ± 4.2	3.9 ± 4.5	<0.01	0.87				20.6 ± 5.8	3.0 ± 6.4	<0.01	0.47			
HADS Anxiety	NDC	10.2 ± 4.1	7.3 ± 4.4	2.9 ± 3.6	<0.01	0.81	0.9 ± 0.7	0.19	0.26	8.0 ± 4.6	2.1 ± 4.3	<0.01	0.50	1.4 ± 0.8	0.10	0.33
	DC	9.5 ± 4.4	7.5 ± 3.4	2.0 ± 3.2	<0.01	0.62				8.7 ± 3.4	0.8 ± 4.1	0.22	0.19			
HADS Depression	NDC	7.2 ± 4.1	5.1 ± 3.8	2.0 ± 3.6	<0.01	0.56	0.2 ± 0.7	0.79	0.05	4.9 ± 4.3	2.3 ± 4.3	<0.01	0.53	1.4 ± 0.8	0.09	0.34
	DC	6.6 ± 3.9	4.4 ± 2.7	2.2 ± 3.3	<0.01	0.67				5.8 ± 3.4	0.8 ± 4.2	0.19	0.20			
FS-D	NDC	40.9 ± 9.4	41.2 ± 15.0	0.3 ± 13.0	0.86	0.02	2.6 ± 2.1	0.21	0.24	39.2 ± 17.7	1.7 ± 16.1	0.42	0.02	1.3 ± 2.9	0.66	0.09
	DC	41.6 ± 8.5	44.5 ± 10.1	2.9 ± 7.4	0.01	0.39				41.1 ± 14.1	0.4 ± 13.2	0.82	0.03			
PBNQ	NDC	5.3 ± 1.0	5.4 ± 1.1	0.1 ± 1.1	0.66	0.06	0.0 ± 0.2	0.81	0.05	5.4 ± 1.1	0.0 ± 1.1	0.89	0.02	0.0 ± 0.2	0.85	0.04
	DC	5.5 ± 1.2	5.6 ± 1.0	0.1 ± 0.9	0.41	0.12				5.5 ± 1.2	0.0 ± 1.1	0.9	0.02			
ISI	NDC	14.2 ± 6.0	8.1 ± 5.7	6.1 ± 6.2	<0.01	1.00	2.9 ± 1.1	**0.01**	0.50	8.5 ± 6.2	5.7 ± 5.8	<0.01	0.98	2.9 ± 1.1	**0.01**	0.52
	DC	12.0 ± 5.4	8.7 ± 4.8	3.2 ± 5.2	<0.01	0.63				9.2 ± 5.8	2.7 ± 5.5	<0.01	0.49			
ASKU	NDC	3.6 ± 0.7	3.9 ± 0.7	0.3 ± 0.7	0.01	0.36	0.0 ± 0.1	0.84	0.04	3.9 ± 0.7	0.3 ± 0.8	0.01	0.35	0.2 ± 0.1	0.26	0.22
	DC	3.6 ± 0.7	3.8 ± 0.7	0.2 ± 0.7	0.04	0.32				3.7 ± 0.7	0.1 ± 0.7	0.3	0.16			
FMI	NDC	2.4 ± 0.6	3.1 ± 0.8	0.6 ± 0.7	<0.01	0.86	0.3 ± 0.1	0.05	0.39	3.2 ± 0.8	0.7 ± 0.9	<0.01	0.86	0.4 ± 0.2	**0.03**	0.44
	DC	2.5 ± 0.6	2.9 ± 0.5	0.4 ± 0.7	<0.01	0.50				2.9 ± 0.7	0.4 ± 0.8	<0.01	0.49			
SCS-D	NDC	2.9 ± 0.8	3.7 ± 1.0	0.8 ± 1.0	<0.01	0.76	0.3 ± 0.2	0.13	0.30	3.9 ± 1.1	1.0 ± 1.2	<0.01	0.82	0.5 ± 0.2	**0.02**	0.45
	DC	3.0 ± 0.7	3.5 ± 0.7	0.5 ± 0.8	<0.01	0.67				3.5 ± 0.9	0.5 ± 0.8	<0.01	0.58			

Abbreviations: ASKU, Self-Efficacy Scale—Short Form; BS, Breast-cancer subscale; FACIT-F, Functional Assessment of Chronic Illness Therapy—Fatigue; FACT-B, Functional Assessment of Cancer Therapy—Breast Cancer; FACT-G, Functional Assessment of Cancer Therapy—General; FMI, Freiburg Mindfulness Inventory; FS, Fatigue subscale; FS-D, Flourishing Scale; HADS-D, Hospital Anxiety and Depression Scale; ISI, Insomnia Severity Index; NDC, nature-based oncology day care clinic program; DC, conventional oncology day care clinic program; P_between_, between-group differences (analysis of covariances); P_within_, within-group changes from baseline (paired *t*-test); PBNQ, Perceived Benefits of Nature Questionnaire; PSS-10, Perceived Stress Scale; SCS-D, Self-Compassion Scale; SD, standard deviation; WHO-5, WHO-Five Well-Being Index.

## Data Availability

The data that support the findings of this study are available from the corresponding author upon reasonable request.

## Supplementary Materials

The following supporting information can be downloaded at: https://www.mdpi.com/article/10.3390/cancers15184595/s1, Table S1: Secondary diagnoses; Table S2: Lifestyle. If not otherwise denoted values are reported as mean ± standard deviation; Table S3: Evaluation; Table S4: Type and number of adverse events.

## Abbreviations

CIM	Complementary and integrative medicine
MBM	Mind–body medicine
MICOM	Mind–body Medicine in integrative and complementary Medicine
NBI	Nature-based intervention
PRO	Patient-reported outcome
CONSORT	Consolidated Standards of Reporting Trials
NDC	Nature-based oncology day care clinic program
MBSR	Mindfulness-based stress reduction
DC	Conventional oncology day care clinic program
MICE	Markov Chain Monte Carlo
MCID	Minimum clinical important difference
