# Supplementary material for: Effects of an Integrative Day Care Clinic Program with a Focus on Nature Therapy in a Hospital Park Setting on Quality of Life in Oncological Patients—A Non-Randomized Controlled Study
Source: Cancers (Basel). 2023 Sep 16;15(18):4595. doi: 10.3390/cancers15184595 (PMC10527019; doi:10.3390/cancers15184595)
Supplement: Supplementary file 1 [file cancers-15-04595-s001.zip › cancers-2573985-supplementary.pdf]

Supplementary Material

Table S1: Secondary diagnoses.

**Secondary Diagnoses** n (%)	**Total (n=107)**	**NDC (n=56)**	**DC (n=51)**
**Cardiovascular diseases** n (%)			
Hypertonus	11 (10.3)	4 (7.1)	7 (13.7)
Metabolic syndrome	1 (0.9)	1 (1.8)	0 (0.0)
Aortic stenosis	1 (0.9)	1 (1.8)	0 (0.0)
Intermittent supraventricular tachycardia	1 (0.9)	1 (1.8)	0 (0.0)
Heart failure	1 (0.9)	1 (1.8)	0 (0.0)
Paroxysmal atrial fibrillation	1 (0.9)	0 (0.0)	1 (2.0)
Hypercholesterolaemia	1 (0.9)	0 (0.0)	1 (2.0)
**Respiratory diseases** n (%)			
Allergic asthma	4 (3.7)	4 (7.1)	0 (0.0)
Chronic sinusitis	1 (0.9)	1 (1.8)	0 (0.0)
Chronic bronchitis	1 (0.9)	1 (1.8)	0 (0.0)
Allergic rhinitis	2 (1.9)	1 (1.8)	1 (2.0)
COPD	1 (0.9)	0 (0.0)	1 (2.0)
**Skin diseases** n (%)			
Atopic dermatitis	3 (2.8)	2 (3.6)	1 (2.0)
Psoriasis	4 (3.7)	2 (3.6)	2 (3.9)
Acne inversa	1 (0.9)	0 (0.0)	1 (2.0)
Rosacea	1 (0.9)	1 (1.8)	0 (0.0)
Urticaria	1 (0.9)	1 (1.8)	0 (0.0)
Seborrhoeic eczema	1 (0.9)	0 (0.0)	1 (2.0)
Exanthem	1 (0.9)	0 (0.0)	1 (2.0)
**Neurological diseases** n (%)			
Polyneuropathy	29 (27.1)	20 (35.7)	9 (17.6)
Tinnitus	5 (4.7)	4 (7.1)	1 (2.0)
Hypacusis	3 (2.8)	2 (3.6)	1 (2.0)
Migraine	6 (5.6)	2 (3.6)	4 (7.8)
Urge incontinence	2 (1.9)	1 (1.8)	1 (2.0)
Restless legs syndrome	1 (0.9)	1 (1.8)	0 (0.0)
Taste disorder	1 (0.9)	1 (1.8)	0 (0.0)
Cephalgia	4 (3.7)	1 (1.8)	3 (5.9)

Vocal fold paralysis	2 (1.9)	0 (0.0)	2 (3.9)
Horner's syndrome	1 (0.9)	0 (0.0)	1 (2.0)
Parkinson's disease	1 (0.9)	1 (1.8)	0 (0.0)
**Gastrointerstinal diseases** n (%)			
Haemorrhoids	1 (0.9)	1 (1.8)	0 (0.0)
Imperative urge to defecate	1 (0.9)	1 (1.8)	0 (0.0)
Irritable bowel syndrome	7 (6.5)	4 (7.1)	3 (5.9)
Colitis ulcerosa	1 (0.9)	1 (1.8)	0 (0.0)
Nausea	1 (0.9)	1 (1.8)	0 (0.0)
Exocrine pancreatic insufficiency	1 (0.9)	1 (1.8)	0 (0.0)
Short bowel syndrome	1 (0.9)	1 (1.8)	0 (0.0)
Chronic gastritis	1 (0.9)	1 (1.8)	0 (0.0)
Difficulty swallowing	1 (0.9)	1 (1.8)	0 (0.0)
Diverticulitis	1 (0.9)	0 (0.0)	1 (2.0)
Diarrhoe	2 (1.9)	1 (1.8)	1 (2.0)
**Psychological diseases** n (%)			
Sleep disorder	72 (67.3)	39 (69.6)	33 (64.7)
Fatigue	62 (57.9)	33 (58.9)	29 (56.9)
Depressive episodes	16 (15.0)	7 (12.5)	9 (17.6)
Concentration disorders	20 (18.7)	11 (19.6)	9 (17.6)
Anxieties	27 (25.2)	13 (23.2)	14 (27.5)
Memory problems	5 (4.7)	2 (3.6)	3 (5.9)
Panic disorder	1 (0.9)	0 (0.0)	1 (2.0)
**Orthopaedic diseases** n (%)			
Cervical spine syndrome	8 (7.5)	5 (8.9)	3 (5.9)
Thoracic spine syndrome	3 (2.8)	3 (5.4)	0 (0.0)
Lumbar spine syndrome	10 (9.3)	9 (16.1)	1 (2.0)
Shoulder arm syndrome	3 (2.8)	2 (3.6)	1 (2.0)
Gonarthrosis	6 (5.6)	3 (5.4)	3 (5.9)
Joint pain	16 (15.0)	11 (19.6)	5 (9.8)
Hip dysplasia	2 (1.9)	1 (1.8)	1 (2.0)
Muscle pain	8 (7.5)	5 (8.9)	3 (5.9)
Koxarthrosis	6 (5.6)	3 (5.4)	3 (5.9)
Omalgia	1 (0.9)	1 (1.8)	0 (0.0)
Omarthrosis	2 (1.9)	2 (3.6)	0 (0.0)

Scoliosis	3 (2.8)	2 (3.6)	1 (2.0)
Rhizarthrosis	2 (1.9)	1 (1.8)	1 (2.0)
Osteopenia	6 (5.6)	4 (7.1)	2 (3.9)
Osteoporosis	2 (1.9)	0 (0.0)	2 (3.9)
**Hormonal diseases** n (%)			
Hashimoto's thyroiditis	3 (2.8)	1 (1.8)	2 (3.9)
Hypothyroidism	13 (12.1)	9 (16.1)	4 (7.8)
Graves' disease	1 (0.9)	1 (1.8)	0 (0.0)
Endocrine orbitopathy	1 (0.9)	0 (0.0)	1 (2.0)
Hyperthyroidism	1 (0.9)	0 (0.0)	1 (2.0)
Menopausal syndrome	14 (13.1)	3 (5.4)	11 (21.6)
Endometriosis	1 (0.9)	0 (0.0)	1 (2.0)
**Blood and lymphatic system, second cancer** n (%)			
Chronic lymphatic leukaemia	1 (0.9)	1 (1.8)	0 (0.0)
Lymphoedema	12 (11.2)	7 (12.5)	5 (9.8)
Chronic pain syndrome	4 (3.7)	1 (1.8)	3 (5.9)
Polycythaemia vera	1 (0.9)	1 (1.8)	0 (0.0)
Colon cancer	1 (0.9)	1 (1.8)	0 (0.0)
Thalassemia minor	1 (0.9)	1 (1.8)	0 (0.0)
Hodgkin's lymphoma	1 (0.9)	1 (1.8)	0 (0.0)
Basalioma	3 (2.8)	1 (1.8)	2 (3.9)
Melanoma	1 (0.9)	0 (0.0)	1 (2.0)
Raynaud's syndrome	1 (0.9)	0 (0.0)	1 (2.0)
Monoclonal gammopathy type IgA	1 (0.9)	1 (1.8)	0 (0.0)
Breast cancer	1 (0.9)	0 (0.0)	1 (2.0)
Cervical cancer	1 (0.9)	0 (0.0)	1 (2.0)
Varicosis	1 (0.9)	1 (1.8)	0 (0.0)
Lymphatic drainage disorder	1 (0.9)	1 (1.8)	0 (0.0)

Abbreviations: NDC, nature-based oncology day care clinic program; DC, conventional oncology day care clinic program.

Table S2: Lifestyle. If not otherwise denoted values are reported as mean±standard deviation.

Lifestyle	Week 0 NDC (n=55)	DC (n=44)	Week 12 NDC (n=50)	DC (n=46)	Week 24 NDC (n=48)	DC (n=45)
**Diet**						
Days per week with at least two units of fruit	5.1±1.9	5.4±2.0	5.8±1.7	5.5±2.0	5.9±1.6	5.6±1.9
Days per week on which at least one third of the meal consists of vegetables	4.9±2.0	5.5±1.7	5.5±1.8	5.4±1.4	5.6±1.9	5.4±1.6
Days per week with meat consumption	1.6±1.5	1.3±1.2	1.5±1.3	1.2±1.1	1.4±1.2	1.2±1.2
Days per week with consumption of high-fat foods	1.8±1.4	1.9±1.6	1.6±1.5	1.4±0.9	1.4±1.1	1.6±1.2
Days per month with fish consumption	3.1±2.5	3.2±2.3	3.1±2.3	3.0±2.5	3.4±2.5	3.0±2.4
**Lifestyle**						
Days per week with use of Kneipp hydrotherapy	0.8±1.4	1.0±1.9	2.9±2.4	3.4±2.3	2.5±2.4	2.8±2.6
Use of phytotherapy n (%)	42 (76.4)	39 (88.6)	47 (94.0)	46 (100.0)	45 (93.8)	41 (91.1)
Practising relaxation exercises n (%)	25 (45.5)	27 (61.4)	49 (98.0)	43 (93.5)	40 (83.3)	35 (77.8)
Yoga n (%)	10 (18.2)	14 (31.8)	21 (42.0)	24 (52.2)	16 (33.3)	18 (40.0)
Qi Gong n (%)	4 (7.3)	8 (18.2)	21 (42.0)	14 (30.4)	11 (22.9)	11 (24.4)
Tai Chi n (%)	1 (1.8)	0 (0.0)	2 (4.0)	0 (0.0)	0 (0.0)	1 (2.2)
Meditation and mindfulness exercises n (%)	21 (38.2)	18 (40.9)	45 (90.0)	35 (76.1)	33 (68.8)	29 (64.4)
Progressive muscle relaxation n (%)	8 (14.5)	8 (18.2)	25 (50.0)	16 (34.8)	15 (31.3)	9 (20.0)
Autogenic training n (%)	4 (7.3)	0 (0.0)	6 (12.0)	0 (0.0)	4 (8.3)	3 (6.7)
Other relaxation exercises n (%)	1 (1.8)	2 (4.5)	8 (16.0)	6 (13.0)	3 (6.3)	6 (13.3)
Minutes per day relaxation exercises	13.3±34.5	7.6±11.2	22.1±20.9	20.2±17.8	20.8±32.7	17.3±19.0

**Cigarette/alcohol consumption**						
Average number of alcoholic drinks per week in the last month	2.3±3.3	4.2±13.7	2.0±2.9	1.7±3.2	2.5±3.6	2.0±2.7
Average number of cigarettes per day in the last month	0.1±1.1	1.9±12.1	0.1±0.7	0.3±2.2	0.2±1.4	0.4±3.0
**Sick leave**						
Sick days in the last 3 months	23.3±35.5	18.2±29.1	29.0±38.4	21.4±34.4	16.3±32.3	15.6±32.1

Abbreviations: NDC, nature-based oncology day care clinic program; DC, conventional oncology day care clinic program.

Table S3: Evaluation (5-point Likert scale, 1=not at all/very difficult to 5=very much/very easy; reported as mean ± standard deviation).

**Evaluation**	**Week 0**		**Week 12**		**Week 24**	
	**NDC (n=55)**	**DC (n=44)**	**NDC (n=50)**	**DC (n=46)**	**NDC (n=48)**	**DC (n=45)**
How would you rate the effectiveness of the day care clinic program in increasing quality of life?	4.0±0.6	4.0±0.5	4.4±0.7	4.1±0.8	4.0±0.8	4.0±0.8
How do you think you did in implementing the day care clinic program?	2.8±0.8	2.8±0.7	3.1±1.0	3.0±1.0	3.2±1.1	3.0±0.9

Abbreviations: NDC, nature-based oncology day care clinic program; DC, conventional oncology day care clinic program.

Table S4: Type and number of adverse events.

Type of adverse event	Total	NDC	DC
Rib contusion	1	1	0
Restlessness	1	1	0
Bicuspid aortic valve	1	1	0
Migraine	2	1	1
Foot pain	1	1	0
Knee osteoarthritis	2	1	1
Brittle fingernails	1	1	0
Shoulder strain	1	0	1
Tinnitus	1	0	1
Hearing loss	1	0	1
Arthrosis	1	0	1
Bronchitis	1	0	1
Tension	1	0	1
Anxiety	1	0	1
Cancer recurrence	2	0	2
Supraspinatus tendon rupture	1	1	0
Tiredness	2	1	1
Edema in the chest	1	0	1
Common cold	3	1	2
Endometriosis	1	0	1
Pulmonary Infiltrate	1	0	1
Psychological stress	2	0	2
Burnout syndrome	1	0	1
Thyroid nodule	1	0	1
Nausea	1	0	1
Dejection	1	0	1
Diarrhea	1	1	0
Gastrointestinal symptoms, unspecific	1	0	1
Breast swelling	1	1	0
Weight gain	1	1	0
Wrist fracture and incisor fracture	1	1	0
Leg pain	1	1	0
Snap finger	1	1	0

Knee pain	2	1	1
Cystitis	1	1	0
Flu	1	0	1
Neck pain	1	0	1
Lower back pain	1	0	1
Skin problems	1	0	1
**Total**	47	18	29

Abbreviations: NDC, nature-based oncology day care clinic program; DC, conventional oncology day care clinic program.
